# The effect of high frequency sacral nerve stimulation on lower urinary tract function in awake, healthy animals

**DOI:** 10.1038/s41598-025-10047-5

**Published:** 2025-07-09

**Authors:** Jia Han, Brett Hanzlicek, Dario Cabal, Yaneev Hacohen, Anna Rietsch, Douglas Gunzler, Steve J. A. Majerus, Margot S. Damaser, Dennis J. Bourbeau

**Affiliations:** 1 Case Western Reserve University, Cleveland, OH USA; 2 Louis Stokes Cleveland VA Medical Center, Cleveland, OH USA; 3 Cleveland Clinic Foundation, Cleveland, OH USA; 4 MetroHealth Medical System, Cleveland, OH USA

**Keywords:** Neuromodulation, Bladder, Spinal cord injury, Biomedical engineering, Bladder, Spinal cord diseases

## Abstract

Many individuals with neurological disorders rely on using catheters to empty their bladder. However, catheters are associated with urethral trauma and urinary tract infections. Peripheral nerve stimulation at frequencies of 500 − 10,000 Hz is associated with reduction of muscle contraction without causing fatigue. We hypothesized that high frequency sacral nerve stimulation would be associated with reduced pelvic muscle activity without reduced bladder pressures. We implanted five healthy cats with pulse generators connected to nerve cuff electrodes on sacral nerves S1 and S2. We applied stimulation at frequencies of 20 Hz, 500 Hz, or 10 kHz. We measured bladder pressure using our custom-designed wireless, catheter-free bladder sensor, and measured pelvic floor electromyogram (EMG) as a proxy for urethral sphincter activity. Stimulation at 10 kHz was associated with a lack of increase in peak-to-peak pelvic floor EMG amplitude compared to stimulation at 20 and 500 Hz, which suggested blockade of the nerves that control the pelvic floor muscles. All three stimulation frequencies yielded bladder contractions. High frequency sacral nerve stimulation may reduce pelvic floor activity without decreasing bladder pressure. This approach may enable catheter-free bladder emptying for individuals with neurologically driven urethral sphincter overactivity.

## Introduction

For efficient bladder emptying to occur, the detrusor muscle contracts while, simultaneously, the urethral sphincter relaxes. Following spinal cord injury (SCI), individuals often develop detrusor-sphincter dyssynergia (DSD), which is characterized by the uncoordinated simultaneous contraction of the urethral sphincter and contraction of the detrusor muscle during bladder emptying^1^. Individuals with DSD typically require the use of an intraurethral catheter to push past the urethral sphincter and empty their bladder manually. However, the use of catheters is associated with urethral trauma and urinary tract infections^2,3^. Catheter-free bladder emptying is thus a high priority goal for people with SCI^4^.

Sacral neuromodulation is an FDA-approved, clinically available approach to improve bladder control by stimulating the sacral nerves that innervate the lower urinary tract^5,6^. Though this therapy is indicated for individuals with overactive bladder and not for DSD, it demonstrates the potential feasibility and clinical acceptance of a sacral nerve interface for restoring function of the lower urinary tract. Alternatively, sacral anterior root stimulation has been clinically shown to improve bladder emptying for individuals with SCI and DSD^7,8^. However, in order to manage unwanted reflex urethral sphincter contractions during voiding, an anterior rhizotomy is usually conducted^9^. It may be possible to relax the urethral sphincter during bladder voiding by electrically blocking the sacral nerves that innervate the urethra.

There is a growing body of research demonstrating the potential applications for blocking the conduction of action potentials using high frequency nerve stimulation. Stimulation at 10 kHz has been shown to effectively block nerve conduction. In animal experiments, 10 kHz sciatic nerve stimulation resulted in significantly reduced or abolished proximally-evoked muscle contractions during the applying of block stimulation^10,11^. In response to stimulation, there was an immediate onset response that evoked an increase in muscle contraction, followed by nerve block and decreased muscle contraction. At the cessation of block stimulation, muscle contractions returned to normal and the muscle was not fatigued. It took less than a second to achieve block and the return to normal function following cessation of block was similarly rapid. Similarly, stimulation of sacral nerves at frequencies of 500–600 Hz has been tested in chronic spinalized dogs^12^. This high frequency stimulation was alternated with 30 Hz sacral nerve stimulation, which evoked bladder contraction, in order to achieve efficient bladder emptying and maintain the animals’ bladders for up to 6 months.

We hypothesized that high frequency stimulation of the sacral nerves that innervate the lower urinary tract would relax the urethral sphincter, promote bladder emptying, and not be uncomfortable. To test this hypothesis, we implanted five healthy cats with sacral nerve cuff electrodes and measured bladder and pelvic floor function in response to sacral nerve stimulation at different frequencies. Stimulation testing was conducted under both anesthetized and awake, behaving conditions over a 1-month period.

## Methods

This study was approved by the Cleveland Department of Veterans Affairs IACUC (protocol 17-012-CT-19-014) and the Cleveland Clinic IACUC (protocol 2019–2260). All experiments were performed in accordance with relevant guidelines and regulations, and the authors complied with the ARRIVE guidelines.

### Surgical procedures

**Fig. 1:**
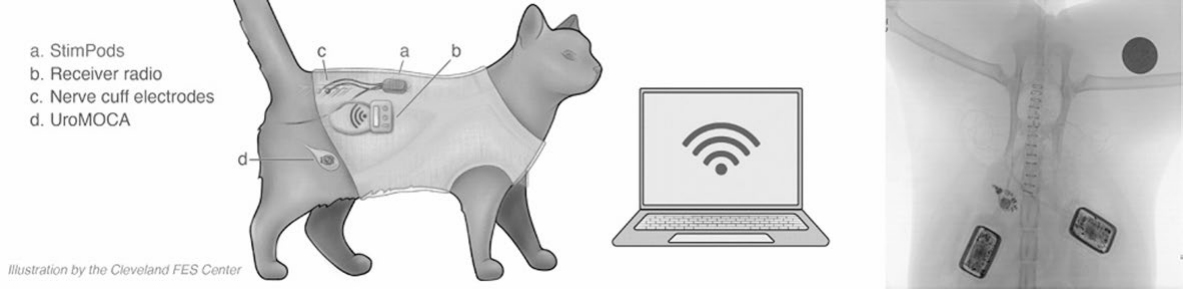
Experimental setup. *Left*: Drawing of experimental setup (left). Animals were implanted with a bladder sensor (UroMOCA), two stimulators (StimPods), nerve cuff electrodes placed bilaterally on the first and second sacral nerves, and EMG electrodes placed bilaterally into the pelvic floor muscle near the anus. Stimulation and data recording were conducted wirelessly with the animal untethered in either an awake, behaving condition or under anesthesia. *Right*: X-ray of implants in subject 4. The dark circle at the top right corner is a United States 25 cent coin, placed in the field for size reference.

In an initial surgery, we implanted *N* = 5 adult cats (2 male, 3 female) with a wireless bladder pressure sensor and wireless stimulation system. Figure 1 depicts the bladder sensor, implanted pulse generator, and electrode implants. First, a sterile 3.5 Fr catheter was placed intraurethrally into the bladder, and an initial urodynamics study was conducted at a fill rate of 2 mL/min of sterile saline under propofol anesthesia (1.0 − 6.0 mg/kg to effect, IV) to confirm normal function of the lower urinary tract. Then, under isoflurane anesthesia (1–3% to effect, inhaled), a midline lower abdominal incision was made to access the bladder, and a cystotomy was performed. We inserted into the bladder a Urological Monitor of Conscious Activity (UroMOCA), which is a wireless, catheter-free bladder sensor that continuously transmits bladder pressure^13^. The bladder incision was closed with monofilament, absorbable suture in a Lembert pattern, and the skin incision was closed with non-absorbable monofilament suture using a simple interrupted pattern. Then we performed a sacral laminectomy, including a midline incision of the lower back from L4-S5 to expose the sacrum and removal of the bone over the dorsal surface of the cauda equina, to expose the sacral nerves. We implanted nerve cuff electrodes on sacral nerves 1 and 2 on the left and right sides, for a total of 4 nerve cuff electrodes (Fig. 1). We inserted barb-tipped intramuscular electromyogram (EMG) electrode leads into the muscles of the pelvic floor near the anal sphincter. These sacral nerve cuff and EMG electrodes were connected to a pair of implanted pulse generators (StimPods, MicroLeads, Somerville, MA). The StimPods were secured in subdermal pockets on the animals’ left and right sides and all device functions were tested. The dorsal skin incision was closed with non-absorbable monofilament suture using a simple interrupted pattern. Both skin incision sites were injected with Bupivacaine (1.25 mg/kg, SQ) before and after incision. The animals recovered for 1 week before beginning data collection sessions.

### Stimulation testing and data recording

Data were collected when cats were anesthetized with propofol (0.3 mg/kg/min, IV), and when they were awake and behaving. Approximately three times per week during weeks 2, 3, and 4 following the initial implant procedure, we tested sacral nerve stimulation with the animals awake and behaving. We measured bladder pressure and pelvic floor EMG in response to sacral stimulation at each of the four cuff electrodes alone, and at frequencies of 20 Hz, 500 Hz, and 10 kHz. Pelvic floor EMG was used as a proxy for urethral sphincter function because (1) they are both innervated by the pudendal nerves that originate from the sacral nerves and (2) pelvic floor EMG was a feasible measure to record during awake, behaving experiments compared to urethral sphincter pressure. We tested stimulation amplitudes up to a testing limit, which was defined as either the tolerance limit or 500 µA, whichever was lower. The tolerance limit was determined once a week and defined as the amplitude at which animals changed behavior in response to stimulation (e.g. attending to their backside or standing up from a relaxed position). Particular care was exercised to avoid causing pain or discomfort. Stimulation amplitudes were tested in intervals of approximately 20 µA up to 200 µA, then approximately 50 µA up to the testing limit. Stimulation parameters were randomized for all combinations of stimulation frequency, amplitude, and cuff electrode, and stimulation was presented for approximately 5 s followed by at least 10 s rest to avoid fatigue.

On days 14 and 28 following the initial implant procedure, animals were anesthetized with propofol and a urodynamics study was conducted to confirm continued normal function of the lower urinary tract. Then we tested combinations of sacral stimulation amplitude, frequency, and cuff electrode while measuring bladder pressure and pelvic floor EMG. The stimulation parameters matched the parameters tested in the most recent awake, behaving data collection session. Stimulation parameters were randomized, and stimulation was presented for approximately 5 s followed by at least 10 s of rest. On day 28 at the end of the experiment, one animal was euthanized with Euthasol (10 mg/mL, IV); the other four animals were awoken. Of those four animals, one was euthanized (as above) for unrelated health complications and the other three were adopted out after being neutered.

### Data processing and analysis

Bladder pressure was sampled at 10 Hz and EMG was sampled at 21 kHz. StimPods only recorded EMG data when they were actively administering stimulation. For every stimulus, we calculated peak-to-peak EMG and bladder magnitude. To facilitate comparison of EMG responses across recording channels and sessions, peak-to-peak EMG was normalized to the maximum EMG recorded on a given channel for each recording session. Bladder magnitude was defined as the difference between the maximum evoked bladder pressure and the average baseline bladder pressure before stimulation was turned on. Bladder pressure data were visually inspected to determine whether the contractions were stimulation driven. To be classified as responsive to stimulation, bladder pressure must have increased after stimulation started and the magnitude had to be greater than the mean plus twice the standard deviation of the baseline pressure. Thus, our primary outcome measure was a binary outcome of presence or absence of bladder contraction in response to stimulation.

Multilevel modeling was used to analyze the effects of frequency, amplitude, and subject condition (i.e. whether the animal was awake or anesthetized) on the peak-to-peak EMG and bladder contraction outcomes. Random effects for sacral nerves S1 versus S2 and the individual cats were included in the models. Interactions between frequency and amplitude, as well as amplitude and anesthetic condition, were tested in a series of models. Stimulation amplitude was treated as a continuous variable, while subject, stimulation frequency, sacral nerve, and condition were treated as categorical variables. EMG was treated as a continuous output variable in our multi-level model. For determining the effect on bladder response, we used logistic regression. A one-way analysis of variance (ANOVA) was employed to determine if stimulation frequency was a significantly contributing factor to the testing limits and the bladder contraction thresholds. We defined α = 0.05 as indicating a statistically significant difference between groups in all statistical tests. R program (v4.3.3, R Foundation) was used for data cleaning and all statistical analyses.

## Results

**Fig. 2:**
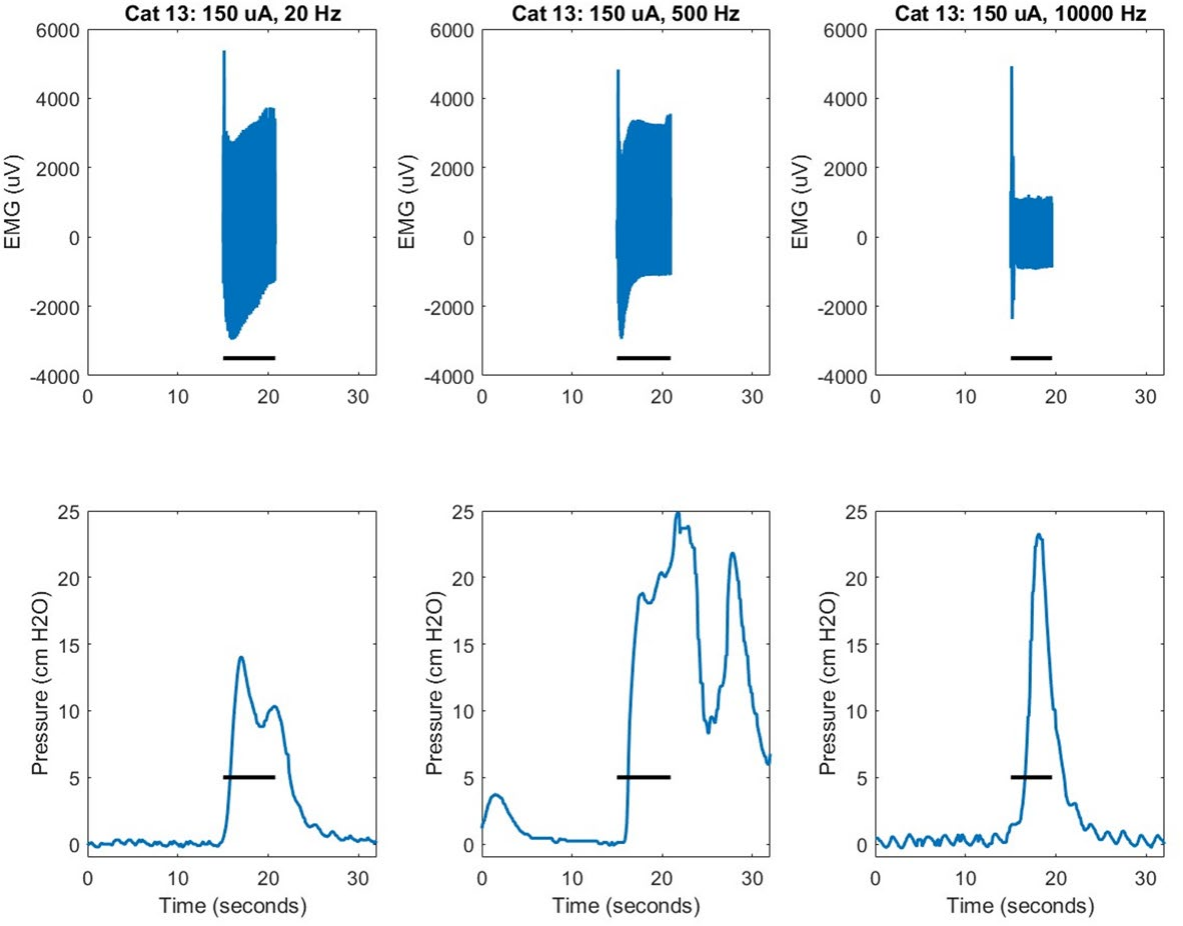
Example EMG and bladder pressure recordings at each stimulation frequency. For these examples, stimulation was 150 uA administered to the first sacral nerve of subject 3 when the animal was awake and behaving. Horizontal black bars denote the administration of stimulation. The UroMOCA recorded bladder pressure continuously during data collection sessions. The StimPods recorded EMG only during the administration of stimulation.

Examples of EMG and bladder pressure data in response to sacral nerve stimulation in awake behaving animals show that EMG amplitude was unstable in the first second of each stimulation cycle, which was an artifact of the stimulation and recording system, but the peak-to-peak amplitude was consistent (Fig. 2). While EMG responses to 20 Hz and 500 Hz showed a constant peak-to-peak amplitude, EMG responses to 10 kHz generally demonstrated an initial high peak-to-peak amplitude for approximately 1 s or less before rapidly reducing to a much lower amplitude. We typically observed clear qualitative observational evidence of contractions of the muscles controlling the pelvic floor, hind limbs, and tail in response to stimulation at 20 Hz and 500 Hz. These responses included anal sphincter contractions that were associated with EMG signals, hind limb extension that was ipsilateral to the stimulated sacral nerve, and tail movement toward the ipsilateral direction of the stimulated sacral nerve. Stimulation at 10 kHz typically resulted similarly in an initial contraction of these muscles (i.e. an onset response) and then relaxation for the duration of the stimulation. We did not observe bladder voiding directly associated with electrical stimulation, whether the animal was awake or anesthetized, at any stimulation frequency or amplitude tested.

Bladder contractions were often, but not always, evoked in response to sacral nerve stimulation, at each of the three stimulation frequencies tested. In response to stimulation, bladder pressure steadily increased at a gradual rate of approximately 10–20 cmH2O/s, which was consistent with the onset of spontaneous (i.e. without stimulation) bladder contractions. Then, when stimulation ceased, the bladder pressure steadily decreased.

**Fig. 3:**
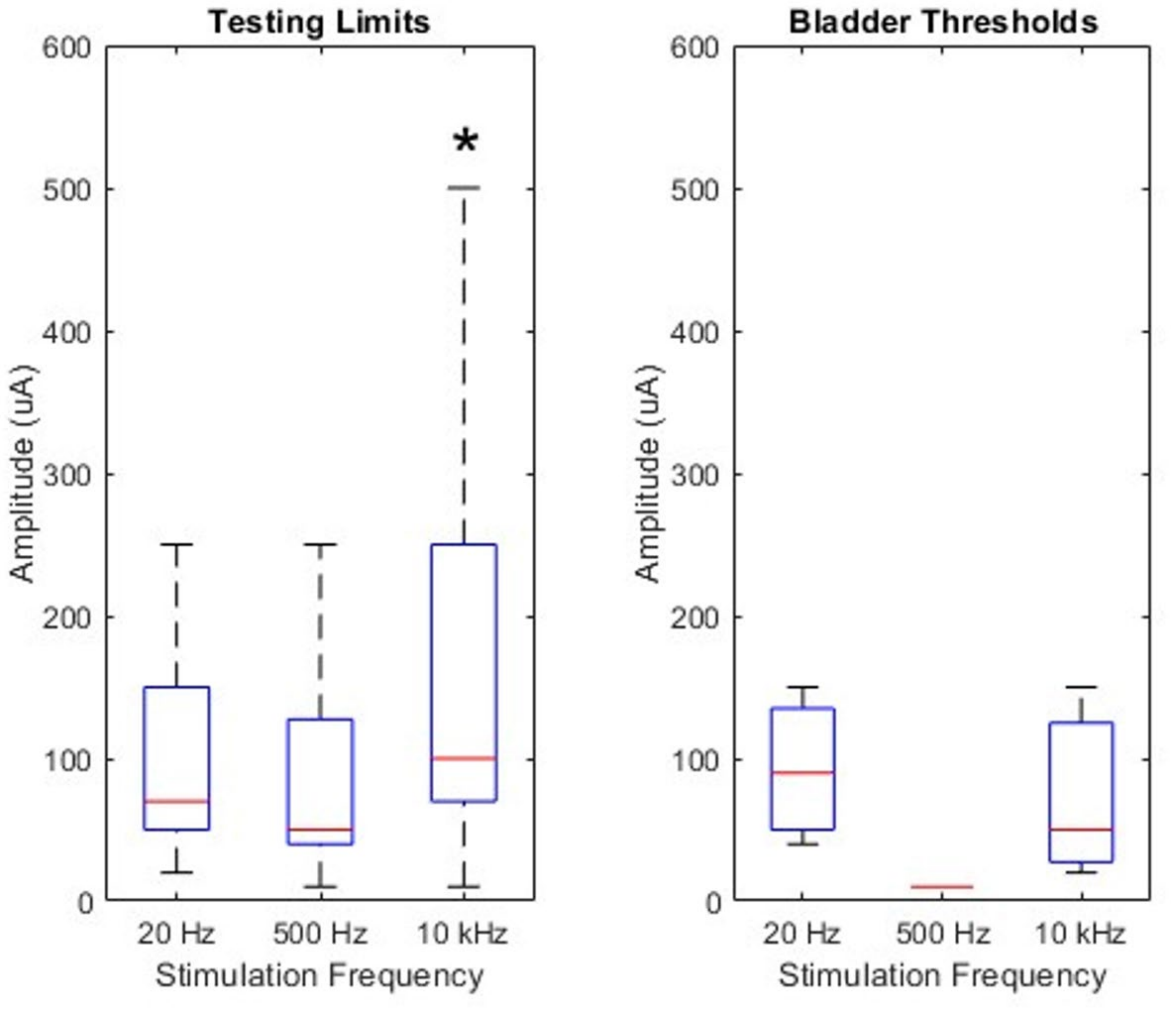
Distributions of testing limits and thresholds to evoke bladder contractions. *Left*: Testing limits were measured once per week each week during awake, behaving data recording sessions. *Right*: Thresholds to achieve a bladder contraction were identified for each animal at each stimulation frequency. On each of the boxplots, the central mark indicates the median, and the bottom and top edges indicate the 25th and 75th percentiles of the data. The asterisk (*) denotes statistical significance.

Once per week, during weeks 2–4 post-implant, we determined the testing limits of each animal at each stimulation frequency and electrode site, for a total of *n* = 177 observations (Fig. 3A). The testing limit was defined as either the tolerance limit or 0.5 mA, whichever was lower. The tolerance limit was defined as the amplitude at which the animal demonstrated behaviors that indicated potential discomfort with the stimulation, such as vocalization or attempting to move away from the stimulus. During awake, behaving data recording sessions wherein stimulation was applied, we did not apply stimulation above these testing limits. Testing limits were predominantly determined by the animals’ tolerance limits. The median testing limits at 20 Hz, 500 Hz, and 10 kHz were 70 µA (20–500 µA), 50 µA (10–500 µA), and 100 µA (10–500 µA), respectively. The testing limit was significantly higher for stimulation at 10 kHz (*p* = 0.0105), suggesting that stimulation at this frequency was more tolerable than at lower stimulation frequencies.

From all recorded cycles of stimulation, we identified the minimum stimulation amplitude that evoked a rise in bladder pressure that was greater than the mean plus two standard deviations of the baseline bladder pressure. Bladder contractions were determined to be evoked by stimulation if this rise in bladder pressure occurred within 3 s after the start of stimulation. We searched for this threshold for each animal and stimulation frequency, for a total of *n* = 9 observations (Fig. 3B). Animal 2 did not show any bladder responses at 500 Hz stimulation, animal 5 did not show bladder responses at 500 Hz or 10 kHz stimulation, and animal 4 did not show bladder responses at any stimulation frequency. The median thresholds at which we observed bladder contractions in response to 20 Hz, 500 Hz, and 10 kHz stimulation were 90 (40–150), 10 (10–10), and 50 (20–150), respectively. While it appears that the threshold to evoke a bladder contraction is lowest when stimulating at 500 Hz, there were an insufficient number of responses to determine if there were significant differences in thresholds as a function of stimulation frequency (*p* = 0.2751).

**Fig. 4:**
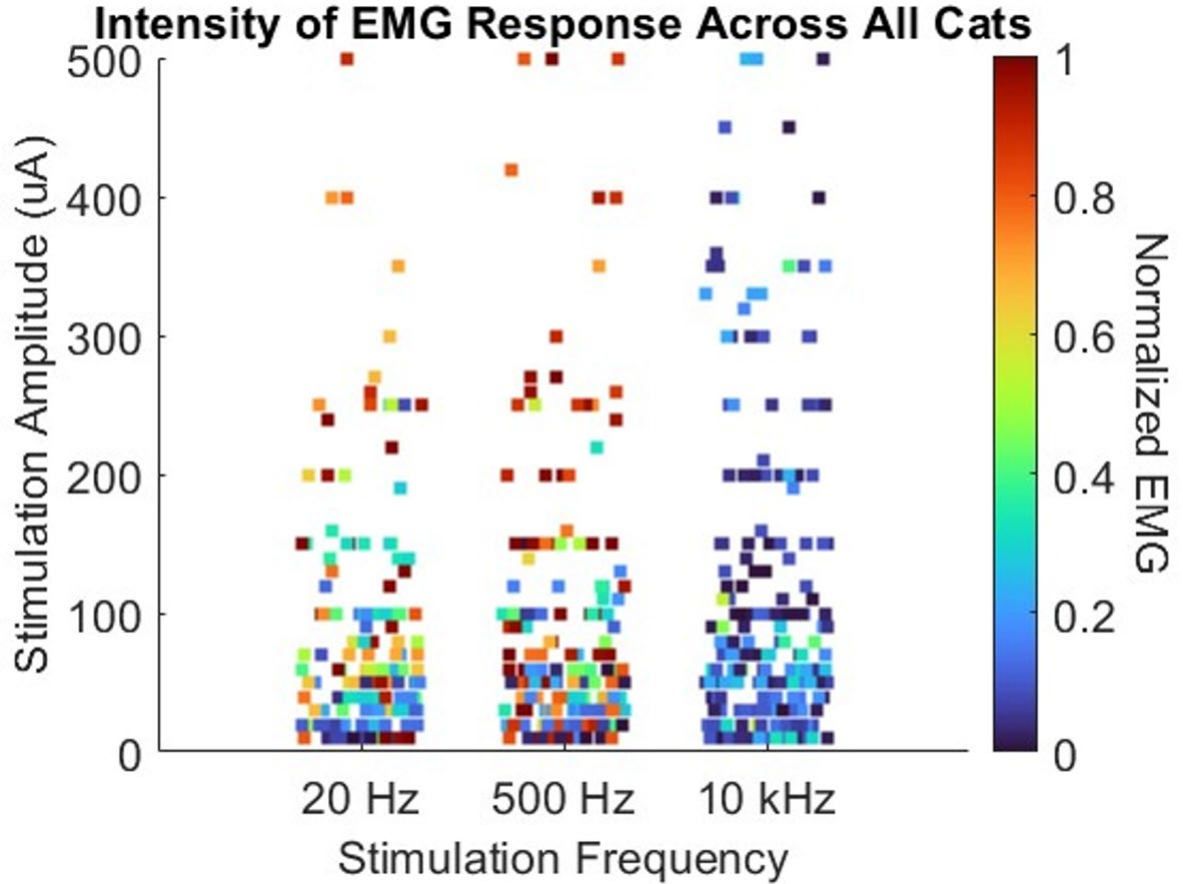
Heat map of EMG responses to sacral nerve stimulation amplitude and frequency. Cooler colors denote lower EMG responses and hotter colors denote higher EMG responses to stimulation. Multiple EMG responses to the same stimulation amplitudes and frequencies are distributed horizontally for visibility.

Figure 4 shows a heat map of the normalized EMG responses to sacral nerve stimulation amplitude and frequency. Cooler colors (i.e. blue) indicate lower EMG responses and hotter colors (i.e. red) indicate higher EMG responses. At 20 Hz sacral nerve stimulation, EMG amplitude increased with increasing stimulation amplitude. We observed this same effect with stimulation at 500 Hz. However, stimulation at 10 kHz did not evoke large EMG responses, regardless of stimulation amplitude. In our multi-level model of EMG response, the interaction between amplitude and frequency was a significant contributor (*p* < 0.001), as were frequency (*p* < 0.001) and amplitude (*p* < 0.001) independently (*n* = 632). The Intraclass Correlation Coefficient (ICC) indicated substantial clustering for animal subject (ICC = 0.25), but not for sacral nerve (ICC < 0.001). Whether the animals were awake or anesthetized was also not statistically significant (*p* = 0.75).

In our model of the presence or absence of a rise in bladder pressure in response to stimulation, the effect of the interaction between amplitude and stimulation at 10 kHz was significant (*p* = 0.01) and negative. The effect of stimulation amplitude alone was significant (*p* = 0.01) and positive. The effects of stimulation frequencies of 500 Hz (*p* = 0.81) and 10 kHz (*p* = 0.70) alone were not more likely to produce a bladder response than stimulation at 20 Hz. Similarly, whether or not the animal was awake or anesthetized did not contribute significantly to our model (*p* = 0.43). The ICC did not indicate substantial clustering for either animal subject or sacral nerve (ICC = 0.01, and ICC < 0.001, respectively).

## Discussion

This study examined the effects of sacral nerve stimulation at different frequencies on bladder and pelvic floor function as a critical step toward an approach using neuromodulation to improve bladder emptying efficiency. Approaches using electrical stimulation of the sacral nerves have been translated into clinical practice to evoke bladder emptying or improve urinary continence. Bladder inhibition for urinary continence can be achieved with low amplitude stimulation of a single sacral nerve, but the mechanism of action remains unclear^14–17^. Bladder excitation can be achieved with higher amplitude stimulation across multiple sacral nerves and this approach includes a posterior rhizotomy to inhibit reflex sphincter activity, which also removes sensation and other reflex activity^18^. Inhibiting urethral sphincter activity without a posterior rhizotomy remains an important clinical translation goal.

This work was conducted in neural intact animals using implanted, wireless devices. Data were recorded under anesthesia and also during animal subjects’ normal daily activities without being tethered, which was more representative of normal physiological behavior. This design allowed us to study the potential effects of anesthesia on our outcome measures, and we found that this effect was not significant. We were also able to observe the effects of stimulation on animal behavior, including bladder emptying behavior and how well the animal subjects tolerated stimulation. We did not observe clear effects of stimulation on voiding behaviors, but we did record rich data on the tolerability of stimulation at the three different frequencies, noting that stimulation at 10 kHz was tolerated at significantly higher stimulations amplitudes compared to stimulation at 20–500 Hz.

We tested sacral stimulation at frequencies of 20 Hz, 500 Hz, and 10 kHz. Stimulation at 20 Hz was chosen because it is associated with activation of motor nerves and tetanic contraction^19,20^ and also associated with inhibition of bladder contractions at low stimulation amplitudes for individuals with overactive bladder^6,21–23^. We hypothesized that stimulation at 20 Hz would be associated with increasing bladder and pelvic floor contractions as stimulation amplitude increased, and we used this stimulation frequency to provide us with a control against which we compared stimulation at higher frequencies. We chose sacral stimulation at 500 Hz because stimulation of the anterior sacral roots in this frequency range has been reported to promote bladder emptying^12^. Finally, stimulation at 10 kHz was chosen because this frequency achieves nerve conduction block^24^. Kilohertz frequency nerve block (KFNB) of sacral nerves could be used to inhibit unwanted urethral sphincter activity because the neurons innervating the urethral sphincter run through the sacral nerves. However, the feasibility and effectiveness of this approach remains unclear because neurons innervating other muscles and organs also run through these sacral nerves (e.g. bladder, colon, anal sphincter, pelvic floor, lower limbs). There is a need to systematically test the frequency space in future work and expand the heat map in Fig. 4. It may be possible to identify an optimal stimulation frequency range wherein urethral pressure is minimized.

We found that stimulation at 20 Hz and 500 Hz evoked both pelvic floor EMG responses and bladder contractions. Stimulation at 10 kHz resulted in significantly reduced pelvic floor EMG magnitudes, regardless of stimulation amplitude. Stimulation generally produced visible muscle contractions, resulting in movement of the animal’s tail and hind limbs. However, in contrast to stimulation at 20–500 Hz, we observed that the tail relaxed within approximately one second of the onset of stimulation at 10 kHz. This lack of response to 10 kHz stimulation provides evidence supporting the hypothesis that stimulation at 10 kHz blocked action potential propagation along the motor efferents and thus reduced motor output.

We chose to apply stimulation for durations of 5 s. These short durations avoided fatigue during data recording sessions that tested a large number of randomized stimulation parameters. We were focused on the responses of the bladder and pelvic floor (i.e. muscle contraction or relaxation), but not necessarily the functional behavioral responses (e.g. bladder emptying). With a 5 s stimulation duration, we did not expect to achieve maximal bladder pressure in response to stimulation. Therefore, our analysis examined the relationship of stimulation on the binary outcome of whether or not a bladder response was observed.

We found that the thresholds for evoking bladder contractions were generally below the testing limits, indicating that stimulation to achieve functional bladder contractions could be tolerable. For stimulation at 20 Hz and 500 Hz, we were increasingly likely to observe a bladder contraction as the stimulation amplitude increased. However, while the effect of stimulation frequency on its own did not significantly contribute to our model of bladder response, the interaction between stimulation amplitude and frequency at 10 kHz was significantly associated with a reduced likelihood of evoking a bladder contraction. It is possible that stimulation at 10 kHz may also block neurons driving a bladder contraction when the stimulation amplitude is sufficiently high. Further work is needed to explore this potential effect, including longer stimulation durations, and to further explore the stimulation parameter space.

Some of the study limitations include the use of healthy neural intact animals rather than animal models of a disease state; the use of pelvic floor EMG as a proxy for urethral sphincter function; lack of abdominal pressure measurement; choice of sacral nerves for stimulation; and short stimulation durations. Our overall goal is to develop an approach using neuromodulation to achieve efficient bladder emptying without catheters or rhizotomy, especially for individuals with spinal cord injury. This experiment tested the effects of sacral nerve stimulation on healthy neural intact animals that did not demonstrate the uncontrolled urethral sphincter reflex contractions that we are ultimately trying to treat. Testing this stimulation in neural intact animals is appropriate at this early stage to first demonstrate that the stimulation modulates function of the lower urinary tract before pursuing more advanced study designs in animals with spinal cord injury.

It was not feasible to continuously measure urethral sphincter function in chronic, awake behaving animals. We used pelvic floor EMG as a proxy for urethral sphincter function. This assumption is reasonable because they are both innervated via the sacral nerves by the pudendal nerves. We did observe anal sphincter contractions that were associated with EMG responses. However, the system only recorded EMG signal during the administration of stimulation, so we did not have EMG data during periods outside of stimulation. Having observed significant modulation of the pelvic floor muscles by sacral nerve stimulation, further research that includes continuous measures of urethral pressure are well justified.

Without a measure of abdominal pressure, there is not a direct method to confirm that the pressures measured in the bladder were predominantly driven by contraction of the detrusor muscle. Bladder pressure increased at a gradual rate that was consistent with the onset of the spontaneous bladder contractions that we observed. This rate is much lower than what would be expected for a pressure response that is due to contraction of abdominal wall muscles. Stimulation often produced significant responses as observed in the EMG and visible movements of the tail and hindquarters, but did not result in changes in bladder pressure. Also, it is unlikely that stimulation of sacral nerves would activate neurons that innervate abdominal wall muscles that could affect bladder pressure measures. Therefore, it is highly likely that our determinations of bladder responses to stimulation are appropriate.

Ultimately, we did not observe bladder voiding in response to sacral nerve stimulation, whether the animal was anesthetized or awake, regardless of stimulation frequency or amplitude. There are several possible reasons that may factor in. One concern with exploring the large parameter space of sacral nerve stimulation in this study was the potential to cause fatigue before the end of our data recording sessions. Therefore, we used short stimulation durations of approximately 5 s. During stimulation, we often observed increases in bladder pressure, but we likely did not observe the peak bladder pressure that could have been achieved for each stimulation amplitude and frequency. We stimulated sacral nerves S1 and S2. It is possible that stimulation of S3 and S4 would be more productive, based on outcomes using the Brindley device^18^. With the animals anesthetized, they may not have been able to void around the intraurethral catheter, and we did not remove the catheter to observe for voiding. With the animals awake, they may have exerted some volitional control to maintain continence. We did not identify an optimum stimulation frequency or systematically sweep through potential stimulation frequencies, so it is possible that there is a frequency range within which we observe an optimal result for bladder emptying.

## Conclusion

Sacral nerve stimulation in awake, behaving animals evoked bladder contractions at stimulation frequencies of 20 Hz, 500 Hz, and 10 kHz. Stimulation at 10 kHz was associated with a significant reduction in pelvic floor activity, consistent with nerve conduction block. Further work is needed to more comprehensively explore the stimulation frequency parameter space, directly measure urethral sphincter pressure responses to stimulation, and determine the effects in an animal model of spinal cord injury and lower urinary tract dysfunction.

## Data Availability

The datasets generated during and/or analyzed during the current study are available in the NIH SPARC Portal repository, https://sparc.science/, or are available from the corresponding author on reasonable request.
